# Targeting Costimulatory Pathways in Systemic Sclerosis

**DOI:** 10.3389/fimmu.2018.02998

**Published:** 2018-12-18

**Authors:** Gonçalo Boleto, Yannick Allanore, Jérôme Avouac

**Affiliations:** ^1^Université Paris Descartes, Sorbonne Paris Cité, INSERM U1016, Institut Cochin, CNRS UMR8104, Paris, France; ^2^Université Paris Descartes, Sorbonne Paris Cité, Service de Rhumatologie A, Hôpital Cochin, Paris, France

**Keywords:** adaptive immunity, inflammation, costimulatory pathways, systemic sclerosis, fibrosis

## Abstract

Systemic sclerosis (SSc) is an autoimmune T-cell disease that is characterized by pathological fibrosis of the skin and internal organs. SSc is considered a prototype condition for studying the links between autoimmunity and fibrosis. Costimulatory pathways such as CD28/CTLA-4, ICOS-B7RP1, CD70-CD27, CD40-CD154, or OX40-OX40L play an essential role in the modulation of T-cell and inflammatory immune responses. A growing body of evidence suggests that T-cell costimulation signals might be implicated in the pathogenesis of SSc. CD28, CTLA-4, ICOS, and OX40L are overexpressed in patients with SSc, particularly in patients with cutaneous diffuse forms. In pre-clinical models of SSc, T-cell costimulation blockade with abatacept (CTLA-4-Ig) prevented and induced the regression of inflammation-driven dermal fibrosis, improved digestive involvement, prevented lung fibrosis, and attenuated pulmonary hypertension in complementary models of SSc. Likewise, potent anti-fibrotic effects were seen with the blockade of OX40L by reducing the infiltration of inflammatory cells into lesional tissues leading to decreased fibroblast activation. Concerning clinical effects, a preliminary observational study suggested some effectiveness of abatacept on inflammatory joint involvement, whereas clinical improvement of skin fibrosis was observed in a small placebo-controlled randomized trial. Currently there is one ongoing phase II clinical trial assessing the efficacy of abatacept in SSc (ASSET trial, NCT02161406). Overall, given the lack of available effective agents and the known toxic effects of immunosuppressive agents approved for use in SSc, costimulatory pathways offer the advantage of a targeted approach to costimulatory signals and potentially a better safety profile.

## Introduction

Systemic sclerosis (SSc) is a rare connective tissue disease characterized by the triad of vascular damage, autoimmunity culminating in widespread fibrosis (1). It can be a devastating disease with a profound impact on life expectancy reflected by high mortality rates (2, 3). The pathogenesis of SSc involves a genetic predisposition together with some partly known environmental triggers. A growing body of evidence suggests that in early stages of the disease there is an interplay between the immune system in particular T and B cells and fibroblasts leading to the perpetuation of the fibrotic process (4).

The activation of naive T cells requires a first signal involving the recognition by the T cell receptor (TCR) of a given antigen and a second non antigen-specific costimulatory signal (5, 6). As a matter of fact, activation and proliferation of naïve T cells are unlikely in the absence of costimulatory signals (7). The CD28-CD80/CD86 pathway is considered the classical co-stimulatory pathway but other pathways such as ICOS-B7RP1, CD70-CD27, CD40-CD154, or OX40-OX40L also play an essential role (8, 9). Negative costimulatory pathways such as CTLA4-B7 or PD1-PDL1/2 play a key role in restraining adaptive immune response (10). There are numerous reports related to the implication of T cell costimulatory pathways in the pathogenesis of several different autoimmune conditions including multiple sclerosis, systemic lupus erythematosus (SLE), and rheumatoid arthritis (RA) (11, 12).

Positive and negative costimulatory signals might be implicated in the pathogenesis of SSc (13, 14). Although a variety of costimulatory molecules have been identified and different immunotherapeutic strategies have been tested, objective clinical responses are rare in SSc patients.

This review aims to discuss the contribution of T cell costimulatory pathways in SSc pathogenesis with a specific focus on their potential therapeutic applications.

## Positive Costimulatory Signals in SSc

CD28, which represents one of the most relevant costimulatory pathways, is essential for naïve T cell activation. Indeed, it promotes T-cell proliferation through the induction of IL-2 secretion after binding to CD80 and CD86 (15). Increased soluble CD28 levels were detected in patients with SSc, compared to healthy subjects, but no correlation was found between soluble CD28 concentrations and extent of skin fibrosis. Furthermore, higher levels of circulating soluble CD28 were more often observed in SSc patients with associated autoimmune disorders (Sjögren's syndrome, systemic lupus, or polymyositis) (12). One of the proposed mechanisms by which CD28 could be implicated in the pathogenesis of SSc is that T cell activation through CD28 is associated with a different profile of cytokine production, with increased proinflammatory and profibrotic cytokines such as TNF, IL-2, IL-6, and IL-10 (16). Moreover, soluble CD28 was shown to inhibit T cell response *in vitro* (12).

The inducible costimulator (ICOS) is a member of the CD28 superfamily. Its structure and function are very similar to that of CD28 (15). ICOS is highly expressed in activated T cells of patients with connective tissue diseases, including RA and SLE (17, 18). ICOS has broad effects on adaptive immune system activation by promoting germinal center formation, T cell proliferation, antibody production and B cell isotype switching (19). Previous reports showed that ICOS serum levels and peripheral T cell expression were increased in patients with early diffuse cutaneous SSc (dcSSc) (20, 21). Overexpression of ICOS in activated T cells induces proinflammatory (IFN-γ, IL-17) and pro-fibrotic (IL-4) cytokine synthesis, promoting fibroblast activation and extracellular matrix synthesis (21).

OX40 and its binding partner, OX40L are members of the TNF receptor superfamily and generate a potent costimulatory signal that upregulates IL-2 production, enhances T cell survival, B cell proliferation, and differentiation and proinflammatory cytokine production (22, 23). OX40 also mediates inactivation of T-reg cell function that unleashes nearby DCs, allowing them to induce an adaptive immune response. OX40 levels were found significantly increased in SSc patients compared to controls and patients with SLE, particularly in the early-onset stage of the disease (24). Two reports confirmed the influence of OX40-ligand (OX40L) polymorphisms in SSc genetic susceptibility, highlighting its role in the disease pathogenesis (13, 25).

Serum levels of the OX40 binding partner OX40 ligand (0X40L) are increased in patients with SSc and were shown to be predictive of the worsening of dermal and lung fibrosis (26). OX40L expression is also prominent in the skin of patients with diffuse SSc. Of great interest, OX40L has been recently reported to be overexpressed in resting and activated dermal fibroblasts, in addition to lesional skin T and B cells. Thus, pathological activation of dermal fibroblasts may be directly mediated by the OX40-OX40L axis, linking directly immunity to fibrosis. The profibrotic effects of OX40L may also be related to its crosstalk with matrix metalloproteinases (MMPs), which are abnormally produced in SSc (27). OX40L has been shown to directly modulate MMP expression in the lesional skin of fibrotic mice invalidated for OX40L (26). Moreover, MMP-2 directly stimulates dendritic cells to up-regulate OX40L on the cell surface (28). MMPs also condition human naïve T cells and dendritic cells to prime TH2 phenotype via an OX40L-dependent pathway (28, 29).

CD40 is another member of the TNF receptor superfamily that plays a pivotal role in mediating a broad variety of immune and inflammatory responses including T cell-dependent immunoglobulin class switching, memory B cell development, and germinal center formation. The binding of CD154 (CD40L) on TH cells to CD40 activates antigen-presenting cells and induces a variety of downstream effects.

A wide array of evidence reported increased CD40 expression in activated CD4+ T cells, skin fibroblasts, and the serum of SSc patients (30–33). The upregulation of the CD40-CD40L axis in immune cells seems at least partly mediated by epigenetic modifications (Demethylation of CD40L regulatory elements) (34, 35).

Soluble CD40L serum concentrations are associated with vascular complications of the disease including pulmonary arterial hypertension (PAH), digital ulcers and destructive peripheral microangiopathy assessed by nailfold videocapillaroscopy (36, 37).

Proteomic analysis of sera from individuals with diffuse cutaneous SSc revealed a multianalyte signature, based notably on CD40L levels, associated with clinical Improvement during Imatinib Mesylate treatment. This results highlights the potential interest of CD40L to predict treatment response in SSc (38).

DNAX accessory molecule 1 (DNAM-1) is an important regulator of the adhesion and costimulation of T cells belonging to the immunoglobulin supergene family (39). Strinkingly, CD226, which encodes DNAM-1, polymorphisms have been identified as a genetic susceptibility factor to SSc, highlighting the contribution of costimulatory pathways in the pathogenesis of this condition (40, 41). DNAM-1 is also overexpressed in the skin of patients with SSc (39) and upregulattion of DNAM-1 in CD8+ T cells is associated with disease severity, suggesting this factor to be a potential therapeutic target in SSc (42).

## Negative Costimulatory Signals in SSc

Since the advent of immunotherapy for the treatment of several neoplastic conditions there has been a rising interest in intrinsic immunity downregulators such as cytotoxic T-lymphocyte-associated molecule-4 (CTLA-4) or programmed cell death 1 (PD-1) and programmed cell death ligand 1 (PD-L1). One of the main drawbacks of immune checkpoint blockade therapy is the emergence of the so-called immune-related adverse events highlighting the role that immune checkpoint play in maintaining immunologic homeostasis (43). Recently there have been few reports of SSc and SSc-like conditions induced by immune checkpoint inhibitors (44, 45).

CTLA-4 is a T cell inhibitory molecule which binds to CD80/86 with higher affinity than CD28, resulting in a drop in IL-2 production and a decreased T cell proliferation (46). Preliminary data suggest that CTLA-4 might contribute to human SSc. Notably, serum soluble CTLA-4 levels (sCTLA4) have been shown to be increased in patients with diffuse cutaneous subset and to correlate with disease severity and activity (14). Increased sCTLA4 serum levels are also observed in several other autoimmune diseases. The biological significance of elevated sCTLA-4 serum levels is not completely clarified yet. sCTLA-4 may specifically inhibit early T-cell activation by blocking CD80/CD86—CD28 interaction. On the other hand, higher levels of sCTLA-4 could compete for the binding of the membrane form of CTLA-4 with CD80/CD86, leading to a reduction in inhibitory signaling (47). In line with this, a meta-analysis of published data showed CTLA-4 polymorphisms conferred susceptibility to SSc (48). Macrophages in particular profibrotic M2 phenotype macrophages may have an important in perpetuating the disease (49). A previous study on tumor immune escape showed that blocking CTLA-4 decreased M2 macrophages differentiation thus suggesting a close relationship between these entities (50).

PD-1 is another inhibitory molecule that regulates T cell tolerance. The expression of PD-1 and its ligands PD-L1 and PDL-2 is antagonized by their soluble forms, leading to augmented T-cell responses (51). Two previous reports showed soluble PD-1 and PD-L1 and PD-L2 to be elevated in SSc patients suggesting it to be correlated to disease development and severity (52, 53). These data seem to suggest that the elevated levels of soluble CTLA-4 and PD-1/PD-L1 and 2 observed in SSc is related to an abnormal T cell and B cell activation.

**Figure 1:**
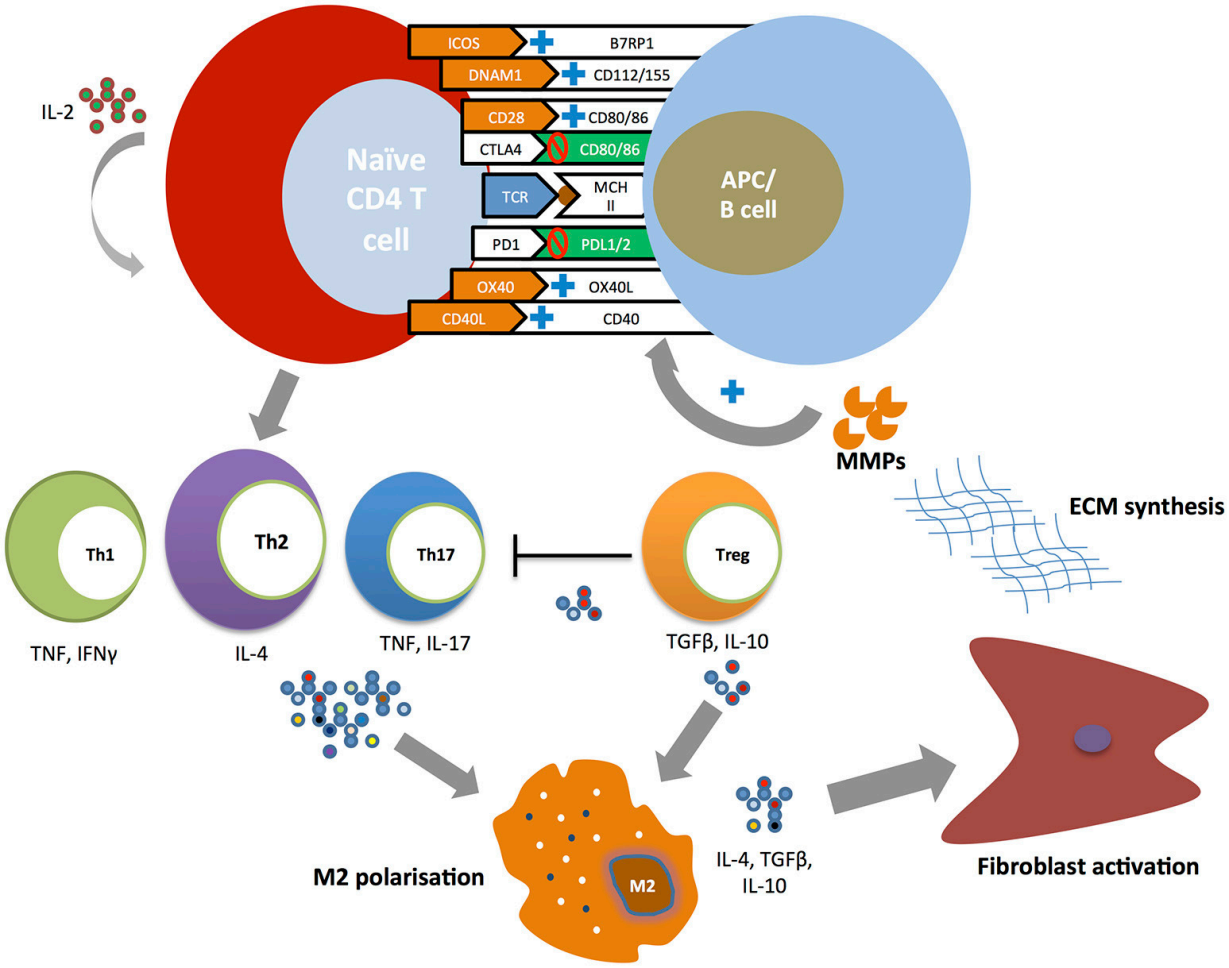
Costimulatory pathways and T cell responses in the pathogenesis of SSc. Naïve CD4+ T cells undergo expansion and differentiation at the time of T cell receptor (TCR) binding to a major histocompatibility complex (MHC) molecule carrying a peptide antigen. The engagement of positive costimulatory pathways (ICOS-B7RP1, DNAM-CD112/115, CD28-CD80/86, CD40-CD154, and OX40-OX40L) and the inhibition of negative costimulatory pathways (CTLA4-CD80/86, PD1-PDL1/2) promote the development of T helper subsets. T helper cell cytokines (TNF, IFNγ, IL-4, IL-17) induce profibrotic type 2 macrophages (M2) polarization with T helper type 2 (Th2) playing a central role. Regulatory T cells (Treg) promote further M2 macrophage polarization and activation through the secretion of IL-10 and TGFβ. M2 activated macrophages promote fibroblast activation leading to excessive extracellular matrix production. In its turn metalloproteinases (MMPs) directly stimulate antigen-presenting cells (APCs) to upregulate positive costimulatory molecules in particular OX40L.

Figure 1 summarizes the putative role of costimulatory pathways in the pathogenesis of SSc.

## Costimulation Therapy—Data From Experimental Models of SSc

Altogether, positive and negative costimulatory T cell signals seem to implicated in the pathogenesis of SSc. Therefore, targeting these pathways through immunotherapy might be more advantageous than current immunosuppressive therapies traditionally used in SSc. Indeed, this strategy offers the hypothetical advantage of targeting the antigen-specific T cells involved in the disease without causing generalized immunosuppression and therefore decreasing the theoretical risk of infectious events (54). Data from *in vivo* complementary animal models give us insight on the effects of the costimulatory pathways blockade as a promising strategy for the treatment of SSc.

A first set of data have shown that DNAM-1 gene invalidation through the use of DNAM-1 deficient mice or the treatment of mice with DNAM-1 neutralizing antibodies prevented the development of dermal fibrosis in the bleomycin mouse model by reducing the infiltration of lesional skin by inflammatory cells and preventing the release of proinflammatory cytokines (TNF-α and IL-6) (39).

OX40L blockade through gene invalidation or targeted therapy using monoclonal antibodies prevented and induced regression of established inflammation-driven dermal fibrosis in the bleomycin mouse model, which mimics early and inflammatory stages of SSc (26). Likewise, OX40L blockade protected against the development of interstitial lung disease and alleviated pulmonary hypertension in the Fra-2 transgenic mouse model, which is characterized by extensive inflammatory infiltrates with features of human vasculopathy, including PH, paralleled by fibrosing alveolitis similar to that in patients with SSc (26). The effects observed with OX40L blockade were mediated by a dramatic reduction of T cells, B cells, and natural killer cells as well as by reduced levels of proinflammatory cytokines such as IL-6 and TNF-α (26). Interestingly, OX40L knockout mice spontaneously develop interstitial pneumonia and severe PH thus addressing several aspects of SSc pathogenesis (24, 55).

Abatacept (CTLA4-Ig) is a recombinant fusion protein comprising the extracellular domain of human CTLA-4 and the modified Fc region of human IgG1 widely used for the treatment of rheumatoid arthritis (56). In complementary murine models of SSc abatacept prevented the development of inflammation-driven fibrosis and reversed established bleomycin-induced fibrosis. Abatacept treatment led to reduced total and activated T cell, B cell and monocyte infiltration in the lesional skin, as well as decreased release of proinflammatory and profibrotic cytokines. Abatacept demonstrated no efficacy in the treatment of late and non-inflammatory dermal fibrosis in the tight skin-1 mouse model, supporting that T cells are necessary to drive the antifibrotic effects of this molecule (57).

Abatacept also improves gastrointestinal involvement in the chronic graft-vs.-host disease (cGvHD) model by decreasing liver transaminase levels and improving colon inflammation. Abatacept alleviated interstitial lung disease and reversed PH in Fra-2 mice by improving vessel remodeling and related cardiac hemodynamic impairment. Abatacept significantly reduced, in lesional lungs of Fra-2 mice, fibrogenic markers levels, T-cell proliferation and M1/M2 macrophage infiltration (58). These findings suggest that abatacept might be an appealing therapeutic approach beyond skin fibrosis for organ involvement in SSc.

## From Bench to Bedside: Data From Observational Studies and Clinical Trials

Data from an observational (59) and case control study (60) suggested beneficial effects of abatacept in patients with SSc. Indeed, in the study from de Paoli et al. (60) abatacept was added to standard therapy in four patients suffering from severe diffuse cutaneous SSc (dcSSc). In this study, abatacept induced a medically significant and pronounced improvement of the modified Rodnan skin score (mRSS) as well as in pulmonary function tests. However, these results are difficult to interpret since regression of skin fibrosis and overall disease activity over time may reflect the natural history of SSc. Data from the EUSTAR (European League Against Rheumatism Scleroderma Trials and Research group) cohort showed that abatacept induced clinical improvement particularly some effectiveness on inflammatory joint involvement on a group of 11 patients with SSc (59).

One small randomized, placebo-controlled trial assessed the efficacy of abatacept in patients with dcSSc over a period of 24 weeks (61). After randomization, 7 patients received abatacept therapy, while 3 patients in the control group received placebo. At week 24, subjects randomized to abatacept showed a trend toward improvement in mRSS (−8.6 *p* = 0.0625). After adjusting for disease duration, mRSS significantly decreased in the abatacept group as compared to the placebo group (−9.8 *p* = 0.0114). Interestingly, after differential gene expression and pathway enrichment analysis the authors showed that improvers tended to be in the inflammatory intrinsic subset at baseline. Notwithstanding, the small sample size does not allow do draw any conclusions regarding its clinical efficacy.

Conversely, pembrolizumab, a PD-1 inhibitor approved for the treatment of advanced melanoma, selected lymphomas, and advanced non-small cell lung cancer due to its robust antitumor immunity, 2 cases of treatment-induced sclerodermoid reactions resembling to SSc have been published (44). This report emphasizes the role of costimulatory pathways and immune checkpoint molecules in the pathogenesis of SSc. A summary of costimulatory pathways in SSc is available on Table 1.

**Table 1:** Summary of the different costimulatory pathway molecules data in SSc.

	**Costimulatory pathway**	**Tissue expression**	**Expression levels**	**Clinical manifestations**	**Experimental blockade in SSc animal models**	**Experimental activation in SSc animal models**	**Clinical trials**	**Main results**
**Positive costimulators**	CD28-CD80/86	Serum	Increased	None	None	None	
	ICOS-B7RP1	Serum, skin	Increased	Early dcSSc	None	None	
	OX40L-OX40	Serum, skin	Increased	Early onset, worsening of dermal fibrosis	Prevented and induced regression of established inflammation-driven dermal fibrosis in the bleomycin mouse model; Protected against interstitial lung disease and pulmonary hypertension in the Fra-2 model	Spontaneous ILD Production of antiDNA antibodies	
	CD40L-CD40	Serum, skin	Increased	Digital ulcers, PH, early/active NVC pattern	None	None	
	CD112/155-DNAM-1	Skin	Increased	Correlates with more severe dermal fibrosis and ILD	None	None	
**Negative costimulators**	CTLA-4-CD80/86	Serum	Increased	dcSSc, correlates with disease activity and severity	None	Prevented induced dermal fibrosis; was effective in the treatment of established fibrosis	1) Pilot study evaluating the clinical and molecular effects of Abatacept in dcSSc 2) Study of Subcutaneous Abatacept to Treat Diffuse Cutaneous Systemic Sclerosis (ASSET) trial (ClinicalTrials.gov identifier: NCT02161406)	1) Trend toward improvement in mRSS 2) Estimated study completion date: September 2018
	PD1-L-PD1	Serum	Increased	Correlates with disease severity	None	None	

CD (cluster of differentiation), ICOS (inducible co-stimulatory molecule), B7RP1 (B7-related protein-1), DNAM-1 (DNAX Accessory Molecule-1), CTLA-4 (cytotoxic Tlymphocyte-associated protein 4), PD1 (programmed death 1), dcSSc (diffuse cutaneous systemic sclerosis), PH (pulmonary hypertension), NVC (nailfold vascular capillaroscopy), ILD (interstitial lung disease), mRSS (modified-Rodnan skin score)

## Research Agenda

SSc is a very severe autoimmune disease that is considered a prototype for studying the pathogenesis of fibrosis in particular the links between fibrosis and immunity (2). Current therapies used in the treatment of SSc remain essentially palliative and do not reverse the natural course of the disease. Given the lack of available effective agents in SSc, and their high toxicity profiles, targeted immunotherapy in particular blocking costimulatory molecules could be a beneficial strategy for SSc and other fibrotic conditions. Hence, in this context, abatacept appears to be a promising therapy for SSc given the encouraging results presented in this review but also given its well-documented safety profile in other rheumatic diseases in particular in RA (62). To better address the issue of abatacept in the treatment SSc patients, the Study of Subcutaneous Abatacept to Treat Diffuse Cutaneous Systemic Sclerosis (ASSET) trial (ClinicalTrials.gov identifier: NCT02161406) is currently ongoing. This study is a randomized placebo-controlled double-blind phase 2 trial of patients with dcSSc comparing subcutaneous abatacept against placebo. The primary outcome of this trial is defined as the change from baseline in the mRSS to month 12. Further randomized-controlled trials assessing the efficacy of costimulation therapy against placebo and standard therapy drugs (p.e. cyclophosphamide, mycophenolate mofetil) are warranted.

## Conclusion

There is a large body of evidence showing that T cell costimulatory pathways play a critical role in the pathogenesis of SSc. Data from *in vivo* experimental animal models and from human studies showed meaningful effects of costimulation blockade in SSc. Of most interest is abatacept a targeted immunotherapy widely used in RA for which a randomized-controlled trial is currently ongoing. Targeted innovative therapies are one of the most important issues in SSc which is a life-threatening condition free of effective therapies. Further trials are awaited enthusiastically by the medical community in order to stop the natural course of this destructive condition.

## Author Contributions

All authors listed have made a substantial, direct and intellectual contribution to the work, and approved it for publication.

### Conflict of Interest Statement

JA has a consultancy relationship and has received research funding in relationship with the treatment of systemic sclerosis from Actelion, Roche, Pfizer, and Bristol-Myers Squibb. YA has a consultancy relationship and received research funding in relationship with the treatment of systemic sclerosis from Actelion, Bayer, Biogen Idec, Bristol-Myers Squibb, Genentech/ Roche, Inventiva, Medac, Pfizer, Sanofi/Genzyme, Servier, and UCB. The remaining author declares that the research was conducted in the absence of any commercial or financial relationships that could be construed as a potential conflict of interest.
